# Electron microscopy and calorimetry of proteins in supercooled water

**DOI:** 10.1038/s41598-022-20430-1

**Published:** 2022-10-03

**Authors:** Jorge H. Melillo, Elizaveta Nikulina, Maiara A. Iriarte-Alonso, Silvina Cerveny, Alexander M. Bittner

**Affiliations:** 1 Donostia International Physics Center (DIPC), Paseo Manuel de Lardizabal 4, 20018 Donostia-San Sebastián, Spain; 2 CIC nanoGUNE (BRTA), Av. Tolosa 76, 20018 Donostia-San Sebastián, Spain; 3 Centro de Física de Materiales (CFM, CSIC-UPV/EHU)-Material Physics Center (MPC), Paseo Manuel de Lardizabal 5, 20018 Donostia-San Sebastián, Spain; 4 Ikerbasque, Basque Foundation for Science, Pl. Euskadi 5, 48009 Bilbao, Spain

**Keywords:** Chemistry, Physical chemistry, Biophysical chemistry, Physics, Condensed-matter physics, Surfaces, interfaces and thin films

## Abstract

Some of the best nucleating agents in nature are ice-nucleating proteins, which boost ice growth better than any other material. They can induce immersion freezing of supercooled water only a few degrees below 0 °C. An open question is whether this ability also extends to the deposition mode, i.e., to water vapor. In this work, we used three proteins, apoferritin, InaZ (ice nucleation active protein Z), and myoglobin, of which the first two are classified as ice-nucleating proteins for the immersion freezing mode. We studied the ice nucleation ability of these proteins by differential scanning calorimetry (immersion freezing) and by environmental scanning electron microscopy (deposition freezing). Our data show that InaZ crystallizes water directly from the vapor phase, while apoferritin first condenses water in the supercooled state, and subsequently crystallizes it, just as myoglobin, which is unable to nucleate ice.

## Introduction

**Figure 1:**
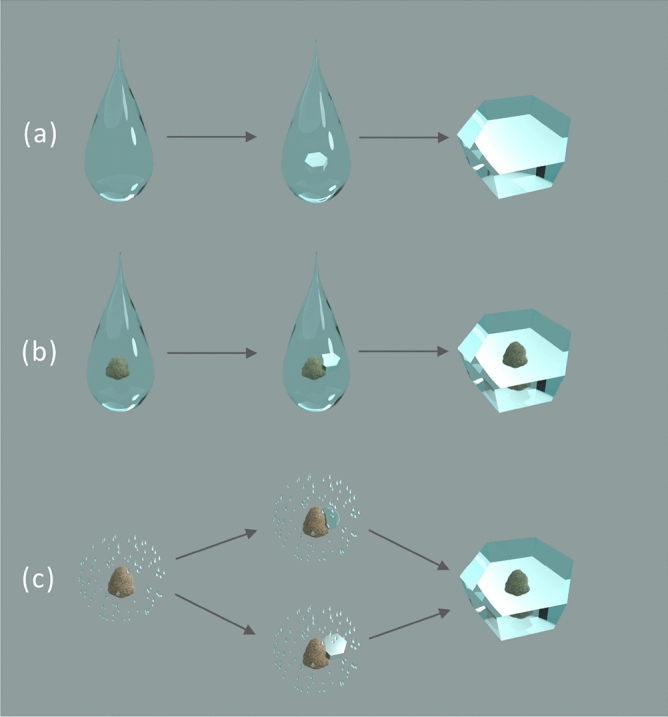
Ice crystallization mechanisms. (**a**) Homogeneous ice nucleation. (**b**) and (**c**) Heterogeneous ice nucleation; (**b**) shows immersion freezing nucleation and (**c**) deposition nucleation. Created with Blender 3.0 (www.blender.org/).

Supercooled water (SCW) is of utmost interest for environmental processes, mainly in the atmosphere^1,2^. Understanding SCW formation is crucial in various technological processes^3–5^, usually to avoid undesired ice formation. SCW at ambient pressure forms below the freezing point, where pure water can be prevented from freezing for long periods, e.g., in hard confinement^6^ or in soft confinement^7–9^. SCW becomes progressively less stable at lower temperatures. In very pure conditions, SCW can remain liquid until − 38 °C^10,11^, which corresponds to homogeneous crystallization, i.e., in this case, ice nucleation starts inside bulk water (see Fig. 1a). For experimentally probing of SCW many methods have been developed, e.g., freezing individual and cascade droplets^12,13^, evaporation from freezing supercooled sessile droplets^14^, condensation from the gas phase (water vapor) on biphilic surfaces^15^, pulsed-laser–heating techniques^10^, and simple optical detection of freezing in ml containers^16^.

The significance of SCW in the atmosphere is mainly related to aerosols in connection with cloud formation. The mechanism by which water vapor condensates to form ice clouds has been investigated for decades because it is essential for understanding cloud formation, which in turn is one of the decisive factors in climate and weather^17^. Merely cooling water vapor does not produce ice. Rather, ice formation requires overcoming the nucleation barrier. A possible way (depending on weather conditions) is immersion freezing, where a droplet of SCW combines with a solid particle, and subsequently freezes (see Fig. 1b). Typical immersion freezing experiments have been carried out with the Zürich ice nucleation chamber (ZINC)^18,19^, with the portable immersion mode cooling chamber (PIMCA)^20,21^, with the droplet ice nuclei counter Zurich (DRINCZ)^22^, with differential scanning calorimeters (DSCs)^23–25^, and with optical microscopes equipped with various cooling systems^26–29^. The essential feature is always cooling of an aqueous solution or suspension at a controlled rate, typically at ambient pressure, to find the crystallization temperature (T_cryst_). In other words, an aqueous liquid mixture is (super)cooled until it crystallizes.

In contrast to immersion freezing, the deposition nucleation mode starts from water vapor, and the crystallization process can follow two different routes, as shown in Fig. 1c^27,30^. One mechanism is the deposition of SCW on a (solid) particle, followed by ice nucleation and growth, ultimately resulting in a bulk ice crystal^31^. The other one is direct vapor to ice nucleation deposition on the particle. Deposition nucleation with both mechanisms is represented in Fig. 1c. One of the most advanced instruments to study such gas–solid transitions with high temporal and spatial resolution is the environmental scanning electron microscope (ESEM)^32–36^.

Although ice nucleation by immersion and deposition consists of water crystallization on a “foreign” particle, both modes are independent. They can provide different information about the nucleation properties of the particle. For example, a recent study showed that the nucleation ability of the aluminosilicate feldspar and of quartz, two essential aerosol minerals, both based on SiO_2_ lattices, differs between immersion and deposition mode^27^. In fact, it is not even trivial to compare both modes.

**Figure 2:**
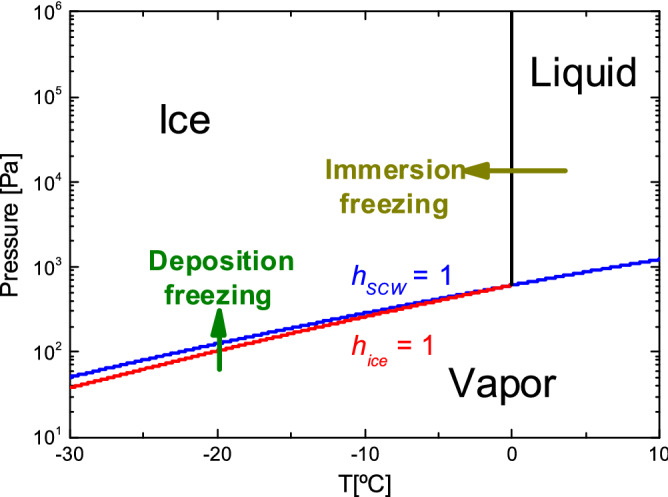
Water phase diagram. The dark yellow arrow corresponds to immersion freezing experiments, and the green arrow to deposition freezing. The red curve indicates the ice saturation pressure (*h*_*ice*_ = 1), and the blue curve the saturation pressure of supercooled water (hSCW = 1). Created with OriginPro 9.0 (www.originlab.com/).

Figure 2 shows the water phase diagram. The liquid-to-ice first order transition corresponds to immersion freezing (dark yellow arrow), and the vapor-to-ice transition to deposition freezing (green arrow), respectively. However, the vapor-to-ice transition can proceed in two ways, represented by the red and the blue curve in the phase diagram (Fig. 2). These curves are based on an improved version of the Clausius-Clapeyron equation 1. The red curve represents the ice saturation pressure *P*_*ice*_, and the blue one is the SCW saturation pressure *P*_*SCW*_, respectively. We can define the relative humidity of ice as:

1 $$h_{ice} = \frac{P}{{P_{ice } }}$$

where *P* is the absolute pressure. Similarly, we can define the relative humidity with respect to SCW as *h*_*SCW*_ by using *P*_*SCW*_ instead of *P*_*ice*_ (see Eq. (1)). When *h*_*SCW*_ ≤ 1 and *h*_*ice*_ ≥ 1, water vapor condensates to ice, and when *h*_*SCW*_ ≥ 1, water vapor forms first SCW and subsequently ice. Nevertheless, it is not always possible to obtain the metastable SCW from deposition freezing; most important, ice nucleation must be suppressed.

Although immersion and deposition freezing modes are different, ice nucleation proceeds in the presence of a “foreign” solid particle. This is usually assumed to be a mineral (inorganic), but can also be composed of organic matter^37^. Recent work on cloud nucleation suggests that bioorganic molecules, such as proteins, play a decisive role^38–42^. On the one hand, this might be surprising because biomolecules had traditionally not been considered as aerosols; on the other hand, ice-nucleating proteins (INPs) are widespread in nature. They induce ice formation in SCW, e.g., the bacterium Pseudomonas syringae^43^ employs the ice nucleation active protein Z (Ina Z) on its outer membrane to trigger freezing at temperatures up to − 2 °C^44^ (ice growth extending *outward* from the bacterial surface is useful to provide nutrients). InaZ is the most efficient and one of the best studied INPs. It comprises ~ 1200 amino acids^45^ with many repeats, which are arranged in a β-helical folding, which in turn is based on stacked β-sheets. The conformation is similar to that of insect antifreeze protein structures^46,47^ (which provide freeze protection *inside* cells). The β-sheets result for both protein types in extended and almost flat surfaces. Further similarities are typical ice-binding sequences (TxT motifs^48,49^) and the ability of arranging the external hydration layer into an ice-like structure^50,51^, despite the opposite function for ice nucleation. However, InaZ has a unique property, namely the capacity to promote energy transfer across the water interface^51^. Moreover, InaZ has direct applications such as freezing technology and food preservation, and especially the induction of snow formation (exploited in the commercial product Snomax)^52^.

There are, however, probably many more protein structures (ternary and quaternary), which can promote ice nucleation. For example, Cascajo-Castresana et al.^16^ recently found that the apoferritin protein cage is a good ice nucleator in immersion freezing experiments. Apoferritin is ubiquitous in many cells from bacteria to vertebrata. It has 174 amino acids, arranged in α-helical conformation, but forms a 24-mer spherical cage (4176 amino acids), which is used by the organisms to store iron. Its ice nucleation ability is apparently not exploited by nature. It could be based on specific properties of the cage surface (hydrophilic, negatively charged, and, compared to small proteins, of relatively small curvature)^53^. Apoferritin has no technical use, but is of great interest for nanoscale science and nanotechnological uses, also in biomedicine.

Myoglobin is a small protein present in high concentrations in vertebrata; its task is oxygen transport. It comprises only 153 amino acids folded into α-helices; its morphology is almost spherical with a high curvature. Myoglobin was the first protein to be characterized by X-ray diffraction. It is often used as a standard example of a globular protein in research; furthermore, it can be envisaged as the “natural color” in red meat.

Here, we present experiments on ice nucleation on the above-mentioned three proteins, in both modes, immersion and deposition freezing. We investigated apoferritin, and chose InaZ as positive control because it is an excellent INP, and myoglobin, which does not nucleate ice, as negative control. Our question is whether the proteins have the same nucleation ability in both nucleation modes. To this end, we analyzed immersion freezing by differential scanning calorimetry (DSC) in solution, and studied deposition freezing by ESEM. The latter allowed us to follow the fate of liquid microdroplets in contact with the (dry) proteins, and in contact with a solid surface (copper). Our results indicate that apoferritin is a good INP in immersion mode but not in deposition mode. Therefore, the two crystallization modes are not correlated.

## Methods

### Sample preparation

Apoferritin (Sigma Aldrich, from equine spleen, batch A3641) was diluted to 3.4 × 10^−4^ g/ml. To remove buffer salts and prevent aggregation of the individual cages, the diluted solution was dialyzed in ammonium bicarbonate (NH_4_HCO_3_) buffer for 96 h. For this purpose, the samples were suspended in 10 mM ammonium bicarbonate (Sigma-Aldrich, 09,830), pH = 7.4 to 7.6, prepared with Milli-Q water. Dialysis was carried out in 10,000 MWCO dialysis cassettes (Thermo Scientific) for 96 h, with the ammonium bicarbonate buffer replaced every 24 h^54^. In the vacuum of the ESEM chamber, NH_4_HCO_3_ completely decomposes into gaseous H_2_O, CO_2_, and NH_3_. The advantage of this procedure is that potential apoferritin disassembly in pure water (pH = 7, extremely low ionic strength) is avoided.

In addition to apoferritin, we employed myoglobin and Snomax solutions as negative and positive controls for ice nucleation, respectively. Snomax is a commercial product that contains (among additives such as carbohydrates and ash) ice-active protein complexes from the bacterium Pseudomonas syringae. This snowmaking additive freezes water at a temperature close to 0 °C. Snomax is therefore considered an excellent INP. We used it without further purification. Myoglobin (Sigma Aldrich, from equine heart) is not known to nucleate ice. Myoglobin was dialyzed in 10,000 MWCO dialysis cassettes (Thermo Scientific) for 24 h in water, replacing it three times during the day, and once overnight. Both Snomax and myoglobin were diluted to 3.4 × 10^−4^ g/ml.

### Immersion freezing and DSC

DSC measurements were carried out on ∼ 10 mg specimens in a Q2000 TA Instruments, operated in standard mode. Sealed aluminum pans were used for all the samples. For the nucleation experiments, the samples were cooled at 1 K/min from 20 to − 33 °C to determine the crystallization temperature. After annealing at − 33 °C for 5 min, the samples were reheated at 10 K/min to determine the melting properties. A helium flow rate of 25 mL/min was used throughout.

### Deposition freezing and ESEM

We employed a Quanta 250 ESEM (FEI, Netherlands), which provides water vapor pressures of > 2000 Pa and sample cooling by a Peltier stage to < − 20 °C. ESEM experiments were carried out in low vacuum mode (pressure limitation 200 Pa) at − 20 °C. This allows the recording of large-scale images (mm size) because a pressure-limiting small aperture at the column cone is not required.

**Figure 3:**
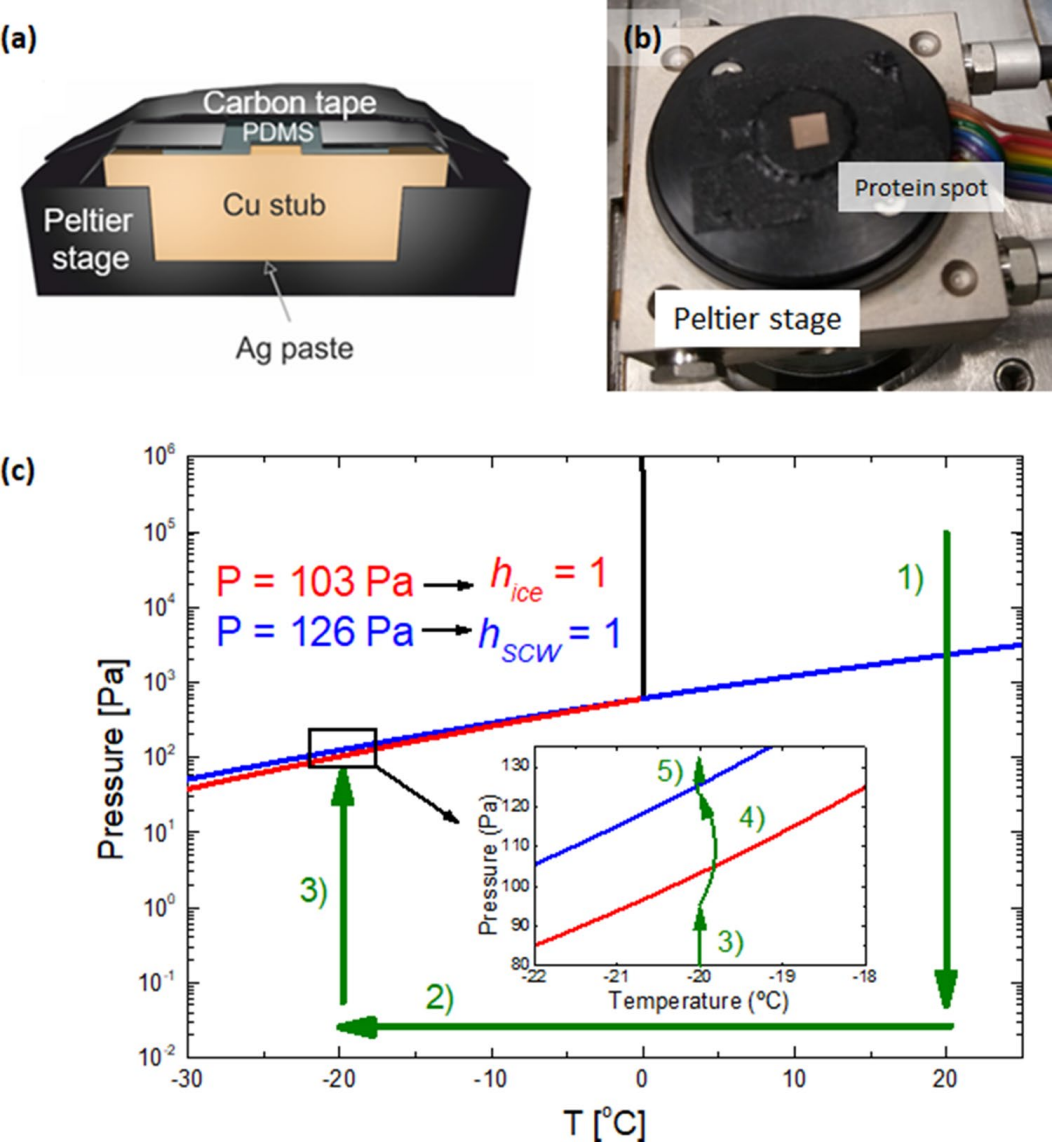
(**a**) Schematic overview and cross-section of Peltier stage prepared for ESEM experiments on SCW (not to scale). (**b**) Photograph of the Peltier stage with the copper stub (substrate surface). The red circle indicates the protein spot. (**c**) Zoom of the phase diagram of water. Green arrows indicate the protocol for deposition freezing experiments. *P* = 103 and 126 Pa correspond to pressure values to reach *h*_*ice*_ = 1 and *h*_*SCW*_ = 1, respectively, at − 20 °C. The numbers refer to the steps of the deposition freezing experiments (see text). Created with OriginPro 9.0 (www.originlab.com/) and CorelDraw (www.coreldraw.com/).

On the Peltier stage, a homemade copper piece (cylindrical stub) was fixed. Heat-conductive silver paste was placed between the stage and the copper piece for optimal heat transfer. The copper surface is the coldest part in contact with the water vapor, hence, condensation starts here. To achieve a highly stable temperature, it is useful to restrict this surface area. To this end, we employed a poly(dimethylsiloxane) (PDMS) film with low thermal conductivity (0.2 Wm^−1^ K^−1^). For decreasing the sample surface area, too, we covered it with carbon tape, mounted above the PDMS film, leaving a window of only several mm^2^. Here, a droplet of protein solution was placed on the copper surface and evaporated in air. This procedure avoids ice crystallization on exposed sample edges^55^. Figure 3a shows a cross-sectional scheme of the complete assembly.

Before every new deposition experiment, the copper stub was polished (final stage with a colloidal suspension of 60 nm SiO_2_ particles) to remove any visible damage, and cleaned in an ultrasound bath, first with acetone, then isopropanol, and finally water. The protein solution was deposited on the upward facing surface of the copper piece by drop-casting, and dried in air (see above). Figure 3b shows the Peltier stage ready for the deposition experiments. The red circle indicates the dry protein spot. It is important to highlight that, whenever ice nucleation occurs, the copper surface should be cleaned, and a fresh protein spot should be prepared. We found ice in ESEM samples even after exposure to high vacuum, suggesting that obtaining a “completely dry” sample is extremely challenging^56^.

In Fig. 3c, green arrows indicate the protocol for deposition experiments in ESEM (note that the pressure is increased by admitting water vapor from a reservoir, which is connected to the chamber through a carefully regulated leak valve):

1. Chamber pressure decrease to high vacuum (< 10^−2^ Pa).
2. Temperature decrease to − 20 °C.
3. Chamber pressure rise to just below *h*_*ice*_ = 1.
4. Pressure jump to just below *h*_*SCW*_ = 1.
5. Pressure jump to *h*_*SCW*_ > 1.

### Characterization of the copper surface

**Figure 4:**
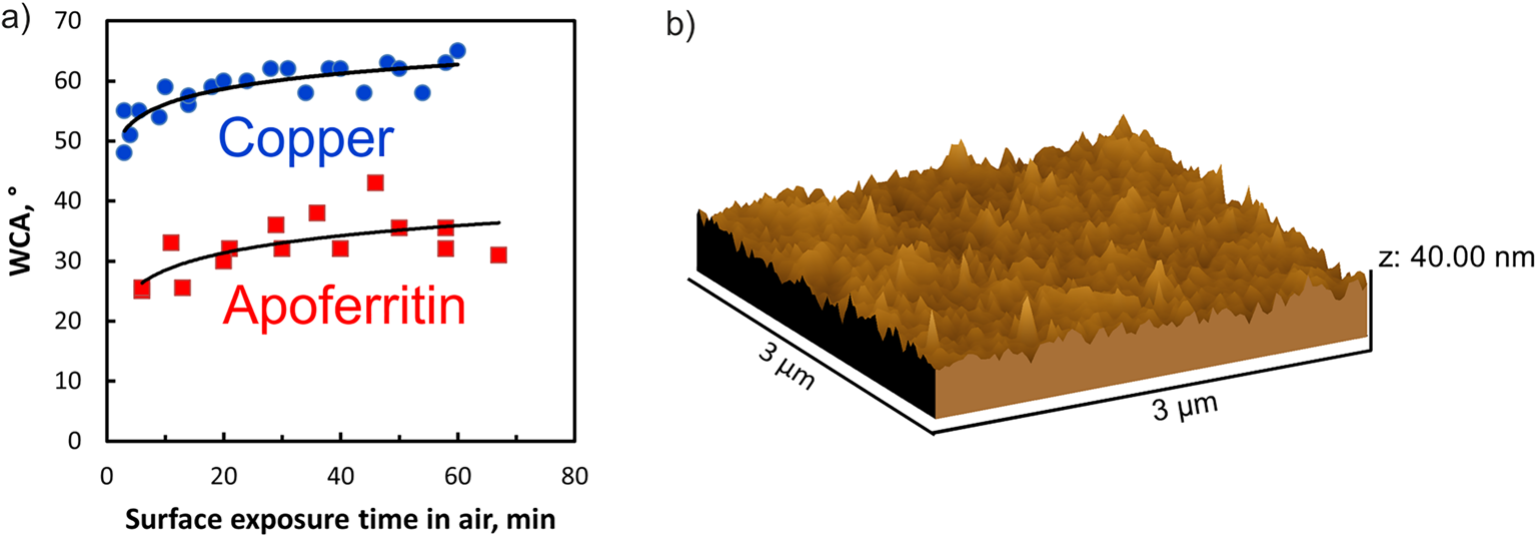
(**a**) Water contact angle as a function of exposure to air, measured on the copper stub surface (blue disks, three independent measurement series) and on an apoferritin droplet dried on copper (red squares, two independent measurement series); (**b**) surface topography measured by AFM. Created with WSXM (wsxm.eu/) and CorelDraw (www.coreldraw.com/).

To characterize the copper surface (which we also call “substrate” in the following, not to be confused with enzyme substrates) in terms of wettability and roughness, static water contact angle (WCA) and AFM measurements were conducted. Figure 4a demonstrates that the copper substrate was more hydrophobic than an apoferritin spot, as seen from the significantly higher water contact angles. Thus, the apoferritin-decorated surfaces exhibit a better wettability than the copper surface, the contact angle on copper is at least 25° higher than that on apoferritin. The dependencies of static water contact angle on surface exposure time in the air, both on copper and apoferritin, are explained by airborne contamination, which generally renders surfaces more hydrophobic over time^57^, compared to freshly prepared surfaces. The effect is clearly present on copper and on apoferritin. The other decisive feature is the roughness. On the mm and microscale, our copper surface has a mirror finish. This is verified by AFM, which shows a roughness of < 20 nm (on a 3 µm × 3 µm area), Fig. 4b. We can infer that there are rather few typical condensation nuclei; once water vapor is condensing, our surface should hinder its immediate freezing. In passing, we note that the substrate is not pure copper; preparation in water and exposure to air translates into the presence of a thin layer of oxide(s).

## Results and discussion

### Water crystallization data from immersion freezing

**Figure 5:**
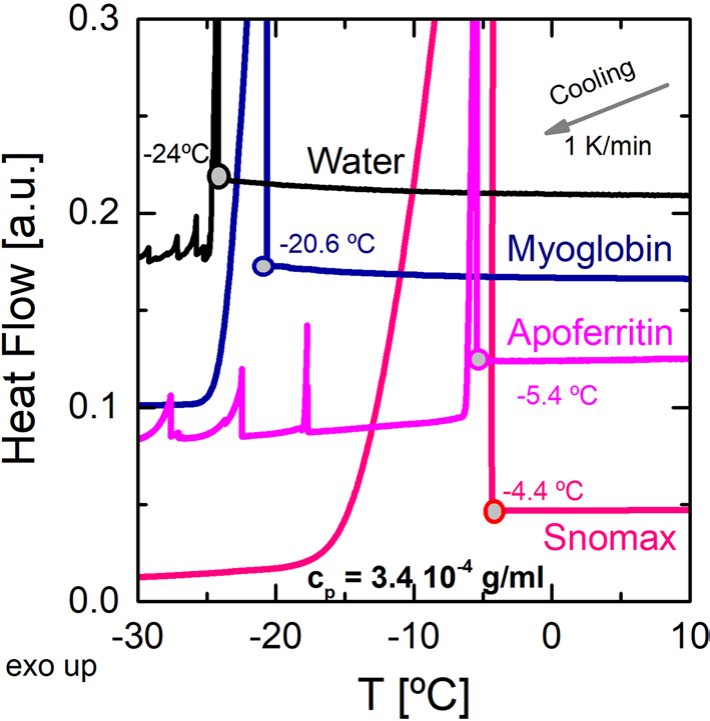
Heat Flow (each curve set off for clarity) as a function of the temperature, for pure water, and for aqueous solutions of apoferritin, Snomax, and myoglobin (all protein concentrations 3.4 10^−4^ g/ml). The crystallization temperatures are indicated in the plot. Apoferritin shows additional crystallization or re-crystallization processes at lower temperatures. Created with OriginPro 9.0 (www.originlab.com/).

This section investigates the crystallization of aqueous solutions of apoferritin, Snomax, and myoglobin in immersion freezing mode. Figure 5 shows the heat flow (HF) as a function of temperature, for pure water and for all the solutions. The protein concentration was in all cases *c*_*p*_ = 3.4 10^−4^ g/ml. Water crystallization is an exothermic process; consequently, heat is released to the surrounding. Therefore, the onset of crystallization is identified as the temperature where the heat flow starts to increase.

As seen in Fig. 5, the onset of crystallization for bulk water was (− 24.0 ± 1.0) °C, measured as an average of 30 independent measurements at a cooling ratio of 1 K/min. Crystallization of pure bulk water is a homogeneous process occurring at ~ − 38 °C^11^. However, the imperfections of a DSC experiment, such as the surfaces of the aluminum pans and possible water impurities, induce heterogeneous crystallization. In such a case, the onset of crystallization is shifted to between − 25 and − 17 °C, depending on the cooling rate^26^. A similar crystallization temperature, (− 20.6 ± 0.9) °C, was found for the myoglobin solution, a protein that does not show any ice nucleation or ice binding properties. This behavior is expected: An aqueous solution of myoglobin contains small globular molecules, which are not aggregated. Their surface is not flat but highly curved: The radius of ~ 2 nm is just above molecular radii, the shape is far from that of a perfect sphere, and each surface spot has its own specific behavior, determined by the local amino acid sequence. This is a significant difference to the proposed requirement of low curvature for INPs^53^.

On the contrary, the crystallization of water with apoferritin occurred at a higher temperature (− 5.4 ± 0.1 °C), and Snomax solutions (containing InaZ) showed (− 4.4 ± 0.1) °C. These temperatures are much higher than those observed in pure water, or in water with myoglobin. This increase in the crystallization temperature is due to the ice nucleation ability of both proteins, as previously reported^12^. Of the many interpretations, we here point out one of the most simple ones, based on a purely geometric effect^58^, which links ice nucleation to flat molecular surfaces. The curvatures of the large protein InaZ, with its extended stacked β-sheets, and of the large supramolecular surface of apoferritin (6 nm radius, almost perfectly spherical) are relatively small. Moreover, both apoferritin and InaZ readily form aggregates^16^, resulting in even lower curvatures. In contrast, the curvature of a small globular protein such as myoglobin (~ 2 nm radius) is very high. Apoferritin showed several additional crystallization or re-crystallization processes at lower temperatures. The released heat was very small, so these processes might not have occurred in all parts of the sample.

In the following, we investigate whether the ability of apoferritin and Snomax to bind ice inside a droplet of supercooled water (i.e., immersion freezing mode) extends to proteins in contact with supersaturated vapor (i.e., deposition mode).

### Water crystallization during deposition freezing on Snomax and myoglobin

**Figure 6:**
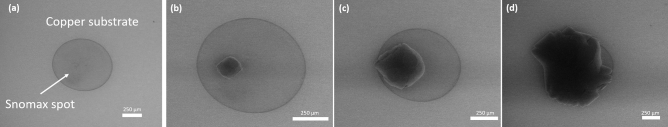
(**a**) Snomax spot on the copper substrate at the condition *P* = 102 Pa and *T* =  − 20 °C (h_ice_ < 1) where no ice condensation is observed. (**b**–**d**) Time series of observing a Snomax spot. Ice growth is triggered on the spot at the condition *P* = 119 Pa, *h*_*ice*_ = 1.05 and *h*_*SCW*_ = 0.94. Figures b–d are images captured during ice growth at (**b**) *t* = 0, c) *t* = 20 s, and d) *t* = 67 s.

We start by analyzing how the crystallization of water is produced on a spot of solid Snomax, which contains InaZ. This protein is considered an excellent ice binding and ice nucleating material, and is known to restrict supercooling to a mere 4.4 K. Therefore, we had expected fast ice condensation on the InaZ spot (we refer to this as a "positive control"). Figure 6a shows the Snomax spot (dark contrast) on the copper substrate under "no ice" conditions (*P* = 102 Pa and *T* = − 20 °C). Ice growth was triggered when the pressure was increased to 119 Pa (Fig. 6b). Subsequently, ice grew exclusively inside the spot (Fig. 6c,d). The experiment shows that ice grows from the rim of the spot (see Fig. SI1 in the supplementary information), and rapidly covers all the substrate. This also proves that ice grows exclusively from the Snomax spot, as no ice nucleation was triggered on the copper outside the protein spot.

Hence, for the InaZ protein (in Snomax), water crystallizes directly from the vapor phase, preventing the formation of SCW. We conclude that InaZ is an excellent INP since it shows an extraordinary ability to avoid SCW in both immersion and deposition modes. This result also confirms our choice of InaZ/Snomax as a positive control.

**Figure 7:**
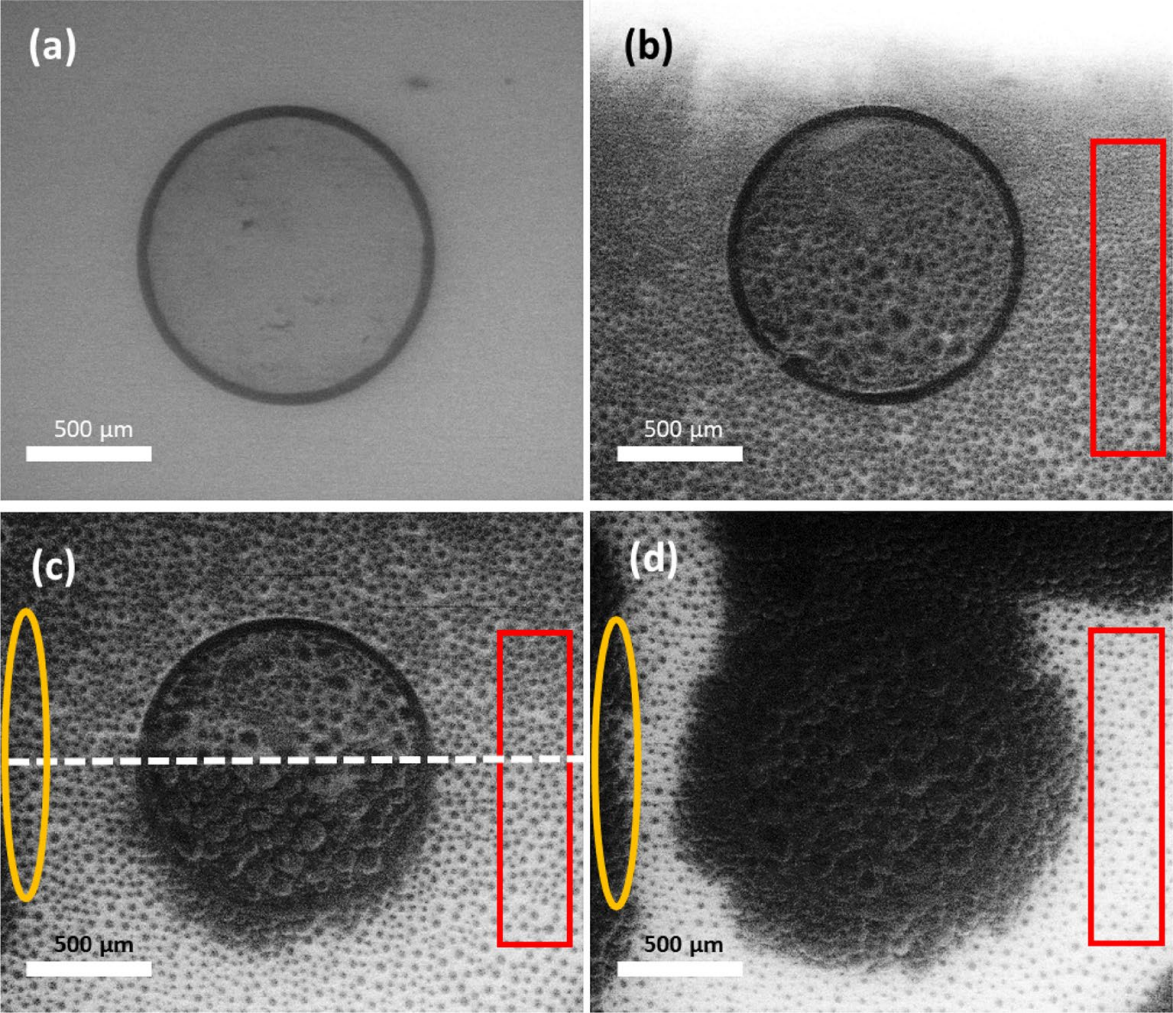
Time-resolved ESEM of the condensation of water vapor, caused by a pressure excursion, on a myoglobin spot on copper. (**a**) *t* = 0 s and *P* = 126 Pa. No ice or SCW are detected. (**b**) *t* = 3 s and *P* = 135 Pa. SCW starts to appear on the protein spot and on the copper substrate. The red rectangle indicates a detail of SCW on the bare copper surface, and the yellow ellipse marks a copper zone free of SCW. (**c**) *t* = 6 s and *P* = 128 Pa. During the scan, SCW evaporates in the red zone. At the same time, ice growth is triggered inside the protein spot (see the dotted white line) and causes crystallization of neighboring SCW droplets by ice bridging. (**d**) *t* = 9 s and *P* = 129 Pa. Ice now covers the complete protein spot. It has also appeared in the yellow area, which is not in contact with the spot. SCW continues evaporating in the red zone. Modified with Power Point (www.microsoft.com/es-es/microsoft-365/powerpoint).

Next, we tested myoglobin, which we had designated as a negative control because the DSC results had shown that it is a relatively poor ice nucleator. Figure 7a shows the myoglobin spot at *h*_*SCW*_ = 1. Nevertheless, we detected neither ice nor SCW. We increased the pressure well above *P*_*water*_, to reach *h*_*SCW*_ > 1. When *h*_*SCW*_ > 1 and when crystallization was not triggered (see Fig. 7b,c above the dashed line), we observed microscale droplets (dark disks) on the substrate and on the myoglobin spot. We interpret these droplets as liquid SCW, but due to the instability of this condition, our temporal resolution was insufficient to analyze the liquid nature of the SCW droplets, e.g., mobility and a changing three-phase line, which we will describe below for the case of apoferritin.

Figure 7c shows that, during the scan, SCW crystallized (see the dashed line). The freezing front propagated very fast inside the protein spot and crystallized all neighboring droplets of SCW, probably by ice bridging^59^. However, ice bridges are not guaranteed to connect to SCW. If an SCW liquid droplet is sufficiently distant from the freezing front, as in the case of the red rectangle in Fig. 7b,d, the SCW liquid droplet can evaporate before an ice bridge can connect^59^. In addition, we have observed a second front from the left of the images (yellow ellipse). This new front was not in contact with the protein spot, so its origin should have been ice crystallization generated on the copper substrate outside of our observation window.

In conclusion, myoglobin is a poor ice nucleator in both immersion and deposition. Notably, in deposition freezing, water crystallization occurs by the first mechanism, i.e., water is condensed in the form of SCW and crystallizes. This is expected, and we can employ our result as a negative control.

### Supercooled water and water crystallization during deposition freezing on apoferritin

**Figure 8:**
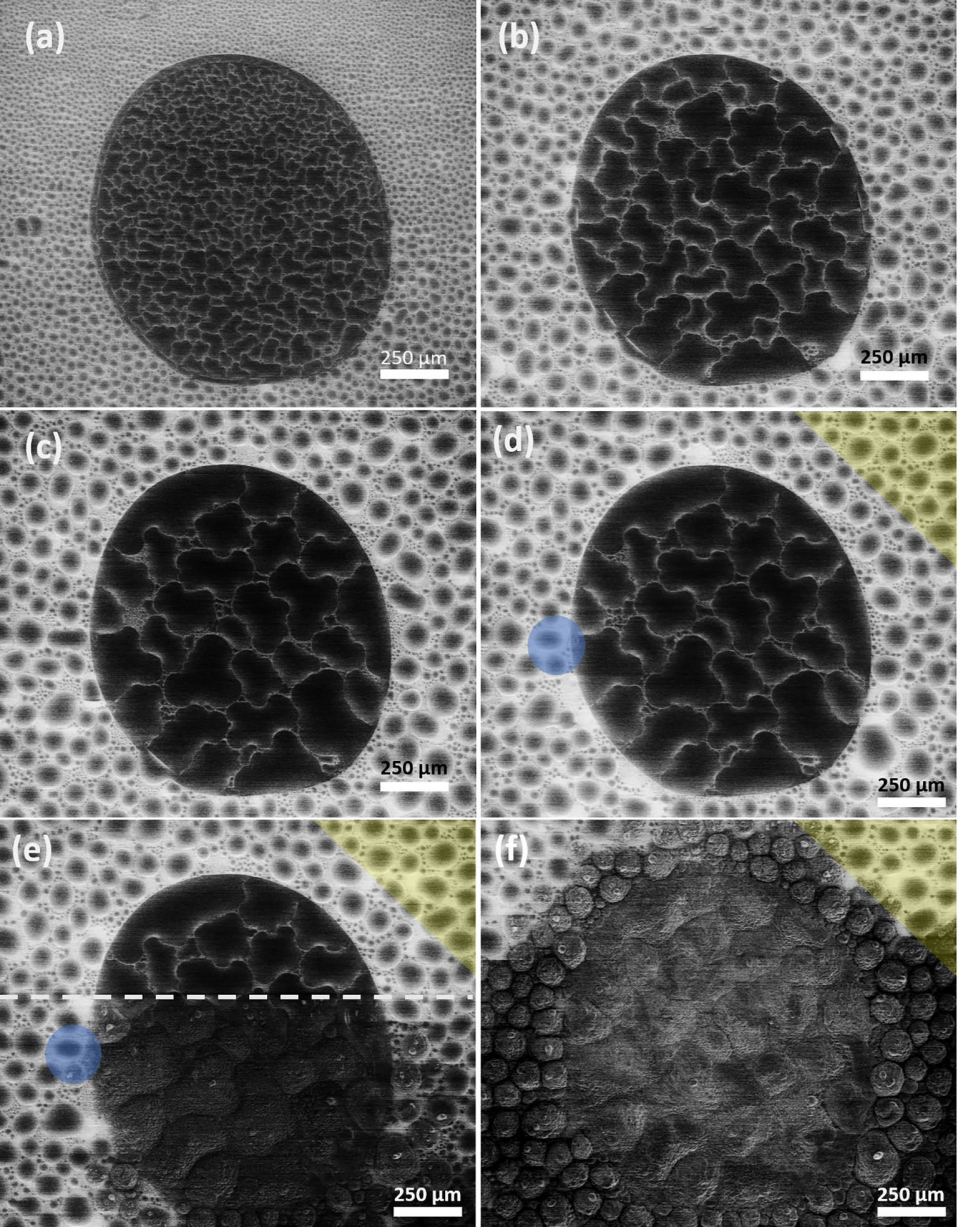
(**a**–**d**) SCW droplets on an apoferritin spot and on the copper substrate. (**a**) *t* = 0 s and *P* = 131 Pa. (**b**) *t* = 2 s and *P* = 134 Pa. (**c**) *t* = 5 s and *P* = 129 Pa. (**d**) *t* = 8 s and *P* = 129 Pa. (**d**–**f**) Crystallization process of SCW. The yellow triangle shows SCW droplets that remain liquid during the crystallization process. The blue disk marks an ice bridging event during freezing front movement. (**e**) *t* = 9 s and *P* = 129 Pa. During the scan, SCW crystallizes rapidly (dashed line) on the apoferritin spot and on some parts of the copper substrate. (**f**) *t* = 10 s and *P* = 129 Pa. Modified with PowerPoint (www.microsoft.com/es-es/microsoft-365/powerpoint).

Figure 8 shows an apoferritin spot under the same conditions as in Fig. 6b. However, we found no ice in this case. By increasing the pressure relatively fast from 119 to 131 Pa (*h*_*SCW*_ > 1), we were able to create droplets (Fig. 8a), which we interpret as SCW. Upon raising the pressure further, from 131 to 134 Pa, the amount of SCW increased (see Fig. 8b). When we decreased the pressure to 129 Pa, with the primary goal to stay closer to *h*_*SCW*_ = 1, the SCW droplets condensated throughout the complete area of the apoferritin spot.

The characteristics of the SCW droplets observed in Fig. 8 can be explained by considering the nature of the surfaces. Since the apoferritin spot represents a hydrophilic surface, SCW liquid droplets spread out and occupy more surface area than outside the spot (on the copper surface). In addition, the shape of these droplets is irregular. Far away from the apoferritin spot we encounter a clean (albeit oxidized) copper surface, which is more hydrophobic (Fig. 4), hence, liquid droplets have a round shape and are smaller in this area. SCW liquid droplets were previously detected on hydrophobic surfaces of similar characteristics^30,58,60^. However, this is to our knowledge the first time that an ESEM experiment has detected SCW liquid droplets on a hydrophilic surface (i.e. on our protein).

Figures 8d–f provide further proof of the liquid nature of the microscale droplets: They move and coalesce while freezing continues. The movement is seen as a changing three-phase line (circumference of a droplet) vapor–liquid–solid. The coalescence of growing ice crystals results in rough (and necessarily solid) surfaces, which we found several seconds later. The video in the supplementary information provides a good visualization of the liquid nature of SCW, and of the ice nucleation process.

Similar to myoglobin, SCW crystallization was triggered on the apoferritin spot during the scan, covering it completely (dashed line in Fig. 8e, the fast ESEM scan direction is horizontal). The freezing front propagated very fast by contact freezing. The water crystallization has furthermore propagated from the apoferritin spot to the remaining bare copper surface by ice bridging. Ice bridging might be present on copper, too, see Fig. 8d,e (compare the blue areas). Altogether, the freezing front propagation is here not instantaneous because the SCW droplets are more isolated. In Fig. 8f, all SCW droplets have crystallized on the apoferritin spot, and almost all on the copper substrate, while in the corner of the figure (yellow triangle) the SCW droplets remain liquid, indicating that the freezing front had started inside the protein spot.

## Conclusions

This work combines calorimetry and real-time environmental scanning electron microscopy (ESEM) measurements to compare ice nucleation in the liquid phase (immersion nucleation) and from the gas phase (deposition nucleation), for three proteins. In addition, it presents electron microscopy of (liquid) supercooled water (SCW).

Myoglobin was tested as a well-known standard protein, for which no ice nucleation action is documented. As expected, this is reflected in the very low nucleation temperature (− 20 °C) in the immersion test. The deposition nucleation from water vapor had not been reported before. We demonstrate the presence of some SCW on the solid protein for a short time (sub-seconds), but in general, the nucleation appears to proceed directly from vapor to solid. Hence, we postulate two crystallization routes: From vapor to solid and from vapor to SCW to solid.

We chose the commercial "snowmaker" Snomax, which contains the ice nucleation protein (INP) InaZ, as test for a well-characterized INP. We reproduced the very high immersion nucleation temperature of − 4.4 °C, which is the base for snow production from SCW. We further demonstrated that InaZ is also an excellent deposition nucleator, resulting in the fast deposition of ice from water vapor onto the solid protein.

Our third protein is the supramolecular cage apoferritin, which is a surprisingly good INP in the immersion mode (− 5.4 °C). We found, however, that the deposition mode involves the formation of SCW from water vapor, and only afterwards crystallization. Hence, apoferritin is a relatively poor deposition mode nucleator.

In comparison, apoferritin and InaZ differ primarily in the deposition mode: Apoferritin is a relatively poor INP for deposition. It develops SCW as an intermediate between gas and solid phase, i.e., first, SCW condensates from vapor, and, with some delay, it freezes. This would be a slow analog to the fast SCW freezing on myoglobin. Another interpretation is that the formation of SCW droplets cannot be avoided on apoferritin; therefore, this protein, although being a good INP, cannot follow the direct vapor route to nucleation of solid ice. Hence, the two crystallization modes are not correlated.

The ESEM observation of SCW also entailed imaging of SCW droplets, whenever they were preserved for sufficient time (minutes). We present a reproducible method to produce SCW, based on a (hydrophobic) polished and solvent-cleaned copper surface, and a water vapor pressure jump at a low temperature. As expected, SCW droplets on our hydrophilic proteins show a very different behavior: They have an irregular shape, cover more area, and feature smaller gaps between droplets, i.e., they nicely wet the surface of a protein spot. Once the crystallization of SCW on proteins is triggered, it is very fast and covers the complete mm-sized protein spot in seconds. The ice propagates from the rim of the spot, where it bridges the protein spot with the copper surface.

Our results demonstrate that ESEM is helpful for experiments in a broad range of water/ice-related topics, specifically for investigating freezing and sublimation on the microscale. This scale should be expanded to the nanoscale, thus providing new insights into the processes leading to ice cloud formation on small nuclei. In addition, the ice growth on airplane wings, a critically dangerous consequence of SCW condensation on flat surfaces, might require investigating submicron surface features. Unfortunately, extending the studies to living cells and biological antifreeze processes, i.e., working with living matter, is not straightforward^61^. Once could circumvent this problem by employing complex biological surfaces such as the cell wall of Pseudomonas syringae^43^, from which InaZ is isolated.

## Supplementary Information

Supplementary Information 1.

Supplementary Video 1.

## Data Availability

The authors declare that all data supporting the findings of this study are available within the paper and its supplementary information files.
