# Supplementary material for: Electron microscopy and calorimetry of proteins in supercooled water
Source: Sci Rep. 2022 Oct 3;12:16512. doi: 10.1038/s41598-022-20430-1 (PMC9529883; doi:10.1038/s41598-022-20430-1)
Supplement: Supplementary file 1 — Supplementary Information 1. [file 41598_2022_20430_MOESM1_ESM.pdf]

Supplementary Information

Electron microscopy and calorimetry of proteins in supercooled water

Jorge H. Melillo, Elizaveta Nikulina, Maiara A. Iriarte-Alonso, Silvina Cerveny, Alexander M. Bittner

Confinement of ice growth to the protein spots

Figure SI1 shows a completely overgrown spot of Snomax. The ice has spread out, but no ice growth is found originating from the copper substrate surface. Figure SI2 proves that apoferritin cannot nucleate ice without a pressure jump above h_{SCW}>1.

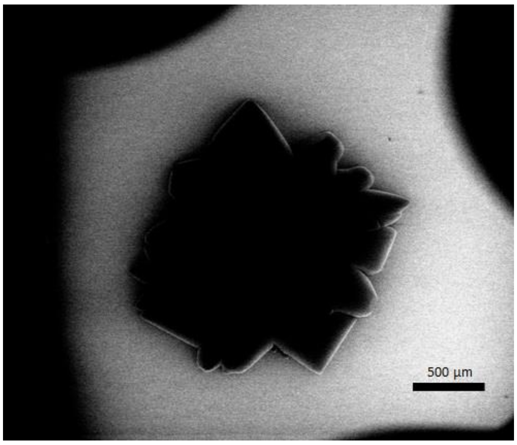

Figure SI1. Detail on ice growth, expanding from a Snomax spot to the complete surface. No ice growth is triggered outside the spot. The dark rims of the image are artifacts.

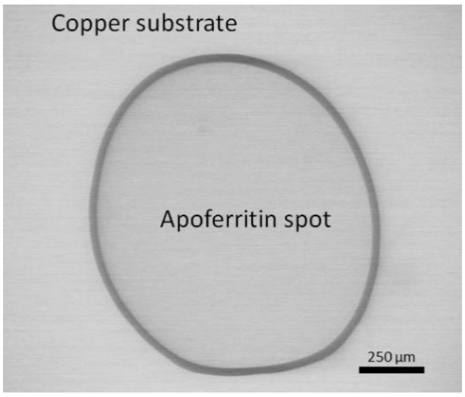

Figure SI2. Apoferritin spot on the copper substrate. P = 119 Pa and T = -20 ºC (h_{ice}>1, but h_{SCW}<1). No ice is observed.

Contamination and purity issues for ice in environmental electron microscopy

Contamination and purity issues are discussed for a similar setup in M. Cascajo-Castresana et al., Atmos. Chem. Phys., 21 (2021) 18629, supplement section 4. The salient points are:

To check for ice surface contamination, we used an area of a flat surface displaying an unusual debris as a reference. Cycles of ice growth and sublimation over this particular area allow for an estimate of the relative amount of contamination, which gave tens of ppm. The contaminants are not acting as nuclei or pinning centers for ice growth.

Protein structures and morphologies in the wet and dry state

InaZ is, in fact, not fully characterized, merely fragments were investigated by XRD, see C.-T. Zee et al., IUCrJ 6 (2019) 197–205. However, these fragments stem from amino acid repeats, which make up a large part of the protein. Hence, the "beta-helical" structure is firmly established. Myoglobin and also apoferritin are staples and even used for resolution tests in XRD. This means that in all three cases, proper crystals were grown. They do contain crystal water (in the case of apoferritin even a very large amount inside the cage). Whether such structures are "dry" or - being grown from solutions – "wet" is a longstanding and complex debate. Given the high stability of myoglobin and especially apoferritin, one can safely assure that the XRD structures are quasi-identical to the structure in solution. This is not clear for InaZ. However, ice nucleators exhibit unusually stable conformations, and specifically stacked beta sheets are not prone to structural changes. Another important point is that structural changes are correlated with high, not low pressure (denaturation by high static pressure), so we do not expect problems in our vapor-filled electron microscope.

Video of freezing deposition on apoferritin

The video "supplSCWapoferritin_video.avi" is based on Figure 8 of the main text. It shows SCW droplets moving and coalescing. When crystallization is triggered on the apoferritin spot, droplet motions remain until the ice front reaches them. The video is based on Figure 8. The conditions are -20°C, and the pressure is first increased from 131 Pa to 134 Pa, which induces SCW, then decreased to 129 Pa to follow ice crystallization.
